# Staff-Identified Palliative Care Needs among Programs of All-Inclusive Care for the Elderly (PACE): A Survey Study

**DOI:** 10.1093/geroni/igaf122.3376

**Published:** 2025-12-31

**Authors:** Molly Nowels, Karolina Sadowska, Mark Unruh, M Carrington Reid, Catherine Riffin, Manali Saraiya, Evan Plys, Daniel Shalev

**Affiliations:** Weill Cornell Medicine, New York, New York, United States; Weill Cornell Medicine, New York, New York, United States; Joan and Sanford I. Weill College of Medicine, Cornell University, New York, New York, United States; Weill Cornell Medicine, New York, New York, United States; Weill Cornell Medicine, New York, New York, United States; New York-Presbyterian Hospital, New York, New York, United States; Massachusetts General Hospital, Boston, Massachusetts, United States; Weill Cornell Medicine, New York, New York, United States

## Abstract

The Program of All-Inclusive Care for the Elderly (PACE) is an interdisciplinary, capitated care model that brings nursing home-level care to dual-eligible older adults living in their own communities. Because serious illnesses are prevalent among PACE participants, PACE programs are federally required to include palliative care within their networks. However, the specific palliative care needs of this unique population have not been characterized, limiting the development of palliative care-PACE integration. To understand PACE staff perspectives on palliative care, we conducted a palliative care needs assessment survey among 81 staff (including CNAs, RNs, OT/SLP/PT, and prescribing providers [MD, NP, PA]) at a PACE program with three sites in New York. Providers were asked if they would like to increase access to palliative care service for their patients, about the most common ways a palliative care specialist could help their patients, and the domains of palliative care in which most of their patients could benefit from additional services. More than half (53%) of respondents agreed that they would like to increase access to palliative care services. Palliative care was seen as most helpful in pain management (80%), caregiver and family support (57%), and psychiatric symptom management (46%). In contrast, respondents identified the most prevalent needs in their patients in caregiver support (69% of patients), advance care planning and goals of care conversations (67%), and psychiatric symptom management (63%). These findings suggest high psychosocial palliative care needs among PACE participants, potentially informing the evolution of uniquely adapted and integrated palliative care services.

